# Early intervention and adding effective doses of EGb761 like *Ginkgo* extract slow down dementia progression: insights to the neurovascular unit

**DOI:** 10.3389/fneur.2023.1240655

**Published:** 2023-12-13

**Authors:** Aynur Özge, Reza Ghouri, Nevra Öksüz, Bahar Taşdelen

**Affiliations:** ^1^Department of Neurology, School of Medicine, Mersin University, Mersin, Türkiye; ^2^Department of Biostatistics, School of Medicine, Mersin University, Mersin, Türkiye

**Keywords:** dementia, acetylcholinesterase inhibitors, memantine, *Gingko* extract, EGb761, mild cognitive impairment, disease progression, trajectory analysis

## Abstract

**Background:**

Dementia is a progressive neurodegenerative disorder characterized by cognitive decline, memory impairment, and functional deterioration. Pharmacological interventions play a crucial role in managing dementia symptoms and potentially slowing down disease progression.

**Objectives:**

This study aimed to investigate the impact of pharmacological interventions, including acetylcholinesterase inhibitors (AChEIs), memantine, and *Gingko* extract, on the progression of dementia, with a specific focus on mild cognitive impairment (MCI), Alzheimer’s disease (AD), and non-Alzheimer dementias.

**Methods:**

A total of 547 participants out of 3,547 cases in a specific dataset followed by the same author, including healthy controls, individuals with MCI, AD, and non-Alzheimer dementias, were included in this study. The follow-up duration was up to 211 months, allowing for a minimum 3 visits comprehensive assessment of disease progression. The treatment approaches included AChEIs, memantine, and combination therapy, with variations in the starting time for these treatments based on the dementia type.

**Results:**

The use of AChEIs and memantine showed efficacy in improving cognitive function and overall function in individuals with MCI, AD, and non-AD dementias. Combination therapy EGb761 like *Gingko* extract with AChEIs and/or Memantine demonstrated a slower progression compared to AChEIs alone in individuals with prodromal dementia (MCI) and AD. The starting time for memantine and combination therapy was earlier in non-AD dementia cases compared to AD dementia cases and prodromal dementia.

**Conclusion:**

Pharmacological interventions, particularly the use of AChEIs and memantine, can have a positive impact on cognitive function and overall function in individuals with dementia. The combination of AChEIs with EGb761 like *Gingko* extract may provide additional benefits in slowing down disease progression in AD cases. Early recognition and accurate classification of MCI subtypes are crucial, and the use of EGb761 like *Gingko* extract is recommended for symptomatic treatment. Future personalized risk predictions based on biomarker constellations may further enhance the multi-target treatment approaches of MCI and different dementia types.

## 1 Introduction

Dementia is a complex neurological disorder characterized by progressive cognitive decline, memory loss, and impaired functional abilities. It is a major cause of disability, socioeconomic burden, and mortality worldwide, and its prevalence is expected to increase dramatically in the coming years due to an aging population. Despite significant advances in our understanding of the pathophysiology of dementia, there is currently no cure for the disease, and management options are aimed at symptom relief and improving quality of life for patients and caregivers (1).

Alzheimer’s disease (AD) is a progressive neurodegenerative disorder that causes dementia, including memory loss and difficulties with thinking and language. The number of people with dementia is expected to triple by 2050, with the incidence increasing significantly with age. Women have a higher prevalence of AD compared to men. AD is characterized by the formation of protein structures called plaques and tangles in the brain, leading to the loss of connections between nerve cells and eventually cell death (2). Non-Alzheimer dementia (NAD) refers to a group of neurodegenerative disorders that are distinct from Alzheimer’s disease (AD) in terms of their underlying pathological features, clinical presentation, and progression. NAD encompasses a range of conditions, including vascular dementia (VaD), frontotemporal lobar degeneration (FTLD) including frontotemporal dementia (FTD), dementia with Lewy bodies (DLB), and Parkinson’s disease dementia (PDD), among others (3). Unlike AD, which is characterized by the accumulation of beta-amyloid plaques and tau tangles in the brain, NAD is associated with other pathological features, such as cerebrovascular disease in VaD and alpha-synuclein aggregates in DLB and PDD (4). The clinical presentation of NAD also differs from AD, with symptoms such as changes in personality, behavior, and language more prominent in FTD, and motor symptoms such as tremors and rigidity more prominent in PDD (3).

Management of dementia is complex, requiring a multidisciplinary approach that includes both pharmacological and non-pharmacological interventions, as well as supportive care for patients and their caregivers (5). A consensus on dementia management has emerged in recent years, with guidelines emphasizing early diagnosis and evidence-based interventions to optimize patient outcomes. Pharmacological interventions such as acetylcholinesterase inhibitors (AChEIs) and memantine have been shown to improve cognitive function and global function in patients with Alzheimer’s disease (AD) and other subtypes of dementia (6).

Recent studies have shown that early and aggressive treatment with cholinesterase inhibitors and memantine can slow the progression of cognitive decline and improve behavioral symptoms in patients with Alzheimer’s disease (AD) (7). However, the effectiveness of combination therapy, and the optimal timing for initiation of such therapy, remain controversial. Some studies suggest that early initiation of combination therapy may lead to better outcomes in terms of cognitive function and behavioral symptoms (6). On the other hand, other studies suggest that late initiation of combination therapy may be more effective in slowing disease progression and improving quality of life (8). Recent studies have suggested that pharmacological interventions with specific herbal extracts, such as the standardized *Ginkgo* extract 160–240 mg (EGb761), may also be effective in slowing the progression of dementia. A meta-analysis of randomized controlled trials found that EGb761 like effective doses *Gingko* extract was associated with significant improvements in cognitive function and daily living activities, as well as a reduction in the incidence of behavioral and psychological symptoms in patients with dementia (9, 10). Additionally, non-pharmacological interventions such as cognitive stimulation therapy and physical exercise have also been shown to improve cognitive function and quality of life in patients with dementia (5). Consensus guidelines on dementia management also stress the importance of individualized care for patients, with care plans tailored to their unique needs and preferences. This patient-centered approach considers the patient’s clinical profile, cultural background, social support, and economic situation (5). However, despite these advances, there is still a significant unmet need for effective and sustainable management options for dementia. Further research is needed to identify novel treatment approaches and to optimize current management options for individuals with dementia. In conclusion, the natural history of dementia is a complex and multifaceted process, and current management options can significantly impact disease progression and quality of life for patients and caregivers.

In this paper, our objective was to examine the impact of pharmacological interventions on the progression of dementia, specifically focusing on acetylcholinesterase inhibitors (AChEIs), memantine, and EGb761 like effective doses *Gingko* extract. These drugs have demonstrated efficacy in enhancing cognitive function and overall function in individuals with Alzheimer’s disease (AD) and other forms of dementia. The primary outcome measures considered in this analysis were changes in cognitive function, assessed through neuropsychological tests, and global function, evaluated using activities of daily living (ADL) scales. Secondary outcomes encompassed the influence of each dementia subtype’s natural progression and the effects of these interventions on cognitive and functional symptoms associated with dementia.

## 2 Materials and methods

### 2.1 Data collection and patient selection

Patients for this study were recruited from a dementia database comprising individuals who had sought consultation at the Dementia Outpatient Clinic of the Neurology Department at Mersin University Medical Faculty between 2000 and 2022, under the supervision of the same senior author (AO). The diagnoses of non-Alzheimer’s dementias adhered to specific criteria for VaD, FTD, LBD, and PDD. These diagnoses were supported by clinical evaluations, neuroimaging, and biomarker assessments (3–6, 8).

Upon obtaining written approval from the ethics committee and the relevant institutional permissions, the study commenced (decision no: 2023/28, date: 10.03.2023). All patients included in the study underwent comprehensive evaluations performed by the same clinic, under the supervision of a single author (AO), with regular quarterly visits. These visits played a pivotal role in thorough data collection, thus contributing to a comprehensive grasp of disease progression. Neuropsychiatric assessments occurred at alternate visits, resulting in an average frequency of twice a year, and were diligently recorded in the dedicated database.

In addition to routine neuropsychiatric assessments, each patient engaged in several other visits as part of this study, specifically addressing medical or medication-related concerns. Within this framework, the attending physician not only prescribed necessary medications but also ordered pertinent laboratory tests if deemed necessary. These proactive medical interventions ensured a comprehensive evaluation of each patient’s health status and requirements.

A crucial aspect of patient evaluation encompassed a systematic approach to differential diagnosis. Following an exhaustive neurological examination, patients underwent a series of essential laboratory procedures to facilitate accurate diagnosis. Subsequently, each patient underwent a comprehensive neuropsychological examination employing standardized methodologies. Significantly, the electronic data recording system within the Turkish Alzheimer’s Working Group’s electronic database,^1^ developed under esteemed leadership, played a pivotal role in documenting and organizing acquired data.

This comprehensive neuropsychological evaluation spanned various cognitive domains, including functional capacity, cognition, numerical range, calculation, abstraction, Word Memory Test (WMT), Clock Drawing Test (CDT), and the Global Deterioration Scale (GDS). Methodologies for these tests have been detailed previously (11, 12), ensuring consistency and accuracy in the assessment process.

These meticulous evaluation processes, facilitated by standardized assessments and comprehensive data recording, form the bedrock for the robustness and reliability of our findings. Through maintaining consistent and thorough evaluations, our aim was to capture the nuanced trajectories of disease progression among the non-Alzheimer’s dementia cases under investigation.

To differentiate Non-AD Dementias from other causes of dementia, a neuroimaging protocol involving MRI or CT was employed for differential diagnosis. In certain cases, standardized SPECT/PET investigations were conducted when necessary (to assist in the differential diagnosis of various dementia subtypes, limited to clinical cases meeting our social insurance system rules, such as those with a positive family history or encephalopathy).

This study encompassed clinical dementia cases (GDS 3 or higher) with at least three comprehensive evaluation visits, including eligible MRI scans for radiological assessment, and complete biochemical screenings for validation. We included GDS 3 cases (also referred to as mild cognitive impairment) to assess longitudinal disease courses. Exclusion criteria encompassed the presence of known inflammatory, infectious, or immune diseases causing cognitive disturbances, overlapping syndromes (e.g., AD plus vascular dementia, motor neuron disorders), comorbid neuropsychiatric disorders (e.g., epilepsy, previously diagnosed psychotic disorders, dependency), major head trauma, severe renal or hepatic failure, recent severe hemodynamic disturbances (e.g., decompensated heart failure, shock, acute myocardial ischemia), residing in nursing homes or palliative care units, and refusal by patients or their legal representatives to participate in the study. Moreover, patients residing in nursing homes or receiving palliative care were excluded due to legal restrictions. Due to the nature of neuropsychiatric test evaluation, only samples with formal education were included in the analysis. Furthermore, patients who were bedridden or in the advanced stages of dementia were not brought to the outpatient clinic by their caregivers and hence had to be excluded from the study.

### 2.2 Statistical analysis

The study employed descriptive statistics, one-way ANOVA, chi-square tests, and repeated measurements ANOVA. Patient clusters and their characteristics were identified through group-based trajectory modeling. The analysis utilized the STATA Plugin. Further details are outlined elsewhere (12).

This study also aimed to explore the effect of pharmacological interventions on the prognosis of patients with AD. To achieve this aim, we used group-based trajectory models, an approach used for identifying distinctive patient clusters according to their outcome measurements. The outcomes of the model were GDS-I, GDS-II, and GDS-III repeatedly measured at three times (admittance, second visit, last visit), and risk factors (or covariates) were pharmacological interventions.

## 3 Results

**Figure 1:**
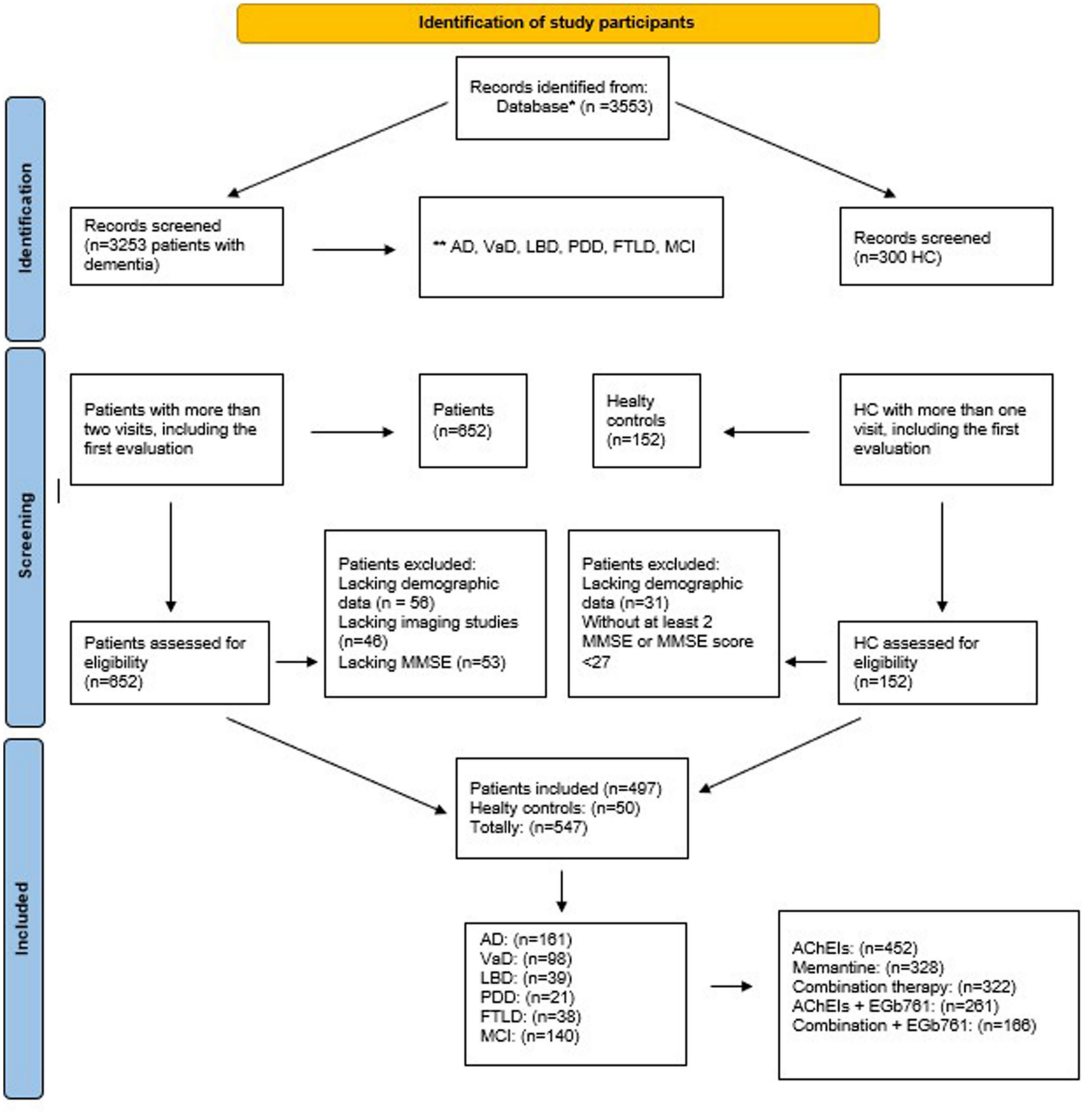
Flow-chart of the study sample. ^*^Epikriz.com (Turkish Alzheimer Database, Mersin Branch). ^**^AD, Alzheimer’s disease; VaD, Vascular dementia; LBD, Lewy body dementia; PDD, Parkinson’s disease with dementia; FTLD, Frontotemporal dementia; MCI, Mild cognitive impairment; MMSE, mini-mental state examination; HC, Healthy controls; AChEIs, Cholinesterase inhibitors; EGb761, Ginkgo biloba extract.

A total of 547 participants out 3,547 samples of our data set were included in this study, comprising healthy controls (*n* = 50), prodromal dementia (n = 140), Alzheimer’s disease (*n* = 161), and non-Alzheimer dementias (*n* = 196; refer to Figure 1).

As shown in Table 1, the mean age of the participants differed significantly among the groups (*p* < 0.001). The healthy control group had a mean age of 65.59 ± 9.96 years, while the prodromal dementia group had a mean age of 70.44 ± 6.94 years. The Alzheimer’s Disease group had a mean age of 73.16 ± 7.23 years, and the non-Alzheimer dementias group had a mean age of 71.02 ± 9.13 years. The duration of follow-up varied among the groups. The first visit to last visit follow-up duration was 55.04 ± 41.98 (min 6 to max 163) months (*n* = 50) for healthy controls, 77.58 ± 44.45 (min 12 to max 203) months (*n* = 140) for prodromal dementia, 71.66 ± 41.98 (min 15 to max 211, *n* = 161) months for Alzheimer’s Disease, and 71.66 ± 41.98 (min 15 to max 211, *n* = 161) months for non-Alzheimer Dementias. Total follow-up duration of study sample was 68.40 ± 39.20 (min 6 to max 211) months. The distribution of gender differed significantly among the groups (*p* = 0.00784) but proportionally distributed between the patients and healthy controls. Most participants in all groups had at least a high school education or above. Most participants in all groups lived with their family rather than alone. There were significant differences in the presentation symptoms among the groups for memory dysfunction (*p* = 0.01848), language dysfunction (*p* = 0.00295), executive dysfunction (*p* < 0.00001), behavioral problems (*p* = 0.00084), self-care problems (*p* < 0.00001), and disorientation (*p* < 0.00001). However, no significant differences were observed for sleep problems (*p* = 0.13841), incontinence (*p* < 0.00001), and loss of appetite (*p* = 0.05862). The prevalence of comorbid medical problems varied among the groups. No significant differences were found in hypertension, thyroid dysfunction, diabetes mellitus, coronary artery disease, hyperlipidemia, stroke, current smoking status, non-smoking status, or regular alcohol consumption. However, significant differences were observed in epilepsy (*p* = 0.00005) and extrapyramidal symptoms (*p* < 0.00001). No significant differences were found in family history of dementia (*p* = 0.55337) or family history of vascular disease (*p* = 0.01463).

**Table 1:** Demographic and clinical features of study sample.

	Healthy control *n* = 50 (9.14%)	Prodromal dementia (MCI) *n* = 140 (25.59%)	Alzheimer’s disease *n* = 161 (29.44%)	Non-Alzheimer dementias *n* = 196 (35.83%)	Total *n* = 547	*p*
Age. year (mean ± SD)	65.59 ± 9.96	70.44 ± 6.94	73.16 ± 7.23	71.02 ± 9.13		**<0.001**
**Follow up duration (months. Mean ± SD)**						
First visit	28.32 ± 35.11 (0–120)	27.96 ± 26.67 (3–120)	33.45 ± 28.07 (3–120)	28.93 ± 25.50 (3–120)	29.96 ± 27.57 (0–120)	0.28905
Second visit	-	14.17 ± 12.98 (3–79)	12.50 ± 11.64 (1–68)	10.72 ± 9.90 (3–90)	12.27 ± 11.46 (1–90)	**0.02319**
Last visit	26.72 ± 27.58 (4–127)	35.45 ± 34.51 (2–174)	25.71 ± 18.68 (2–93)	22.92 ± 17.91 (3–94)	27.29 ± 24.76 (2–174)	**0.00005**
**Gender**						
Female *n* (%)	28 (56%)	78 (55.71%)	110 (68.32%)	99 (50.51%)	315 (57.59%)	**0.00784**
Male *n* (%)	22 (44%)	62 (44.28%)	51 (31.68%)	97 (49.49%)	232 (42.41%)	
**Formal education, *n* (%)**						
Basic school	42 (84%)	120 (85.71%)	142 (88.20%)	183 (93.37%)		**0.01886**
Middle school	-	4 (2.86%)	8 (4.97%)	3 (1.53%)		
High school	-	5 (3.57%)	3 (1.86%)	2 (1.02%)		
University	8 (16%)	11 (7.86%)	8 (4.97%)	8 (4.08%)		
**Living**						
Alone	3 (6%)	23 (16.43%)	33 (20.50%)	33 (16.84%)	92 (16.82%)	0.12414
With family	47 (94%)	117 (83.57%)	128 (79.50%)	163 (83.16%)	455 (83.18%)	
**Presentation symptoms, *n* (%)**						
Memory dysfunction		51 (36.43%)	82 (50.93%)	82 (41.84%)		**0.01848**
Language dysfunction		85 (60.71%)	117 (72.67%)	133 (67.86%)		**0.00295**
Executive dysfunction		19 (13.57%)	64 (39.75%)	61 (31.12%)		**<0.00001**
Behavioral problem		2 (1.43%)	16 (9.94%)	7 (3.57%)		**0.00084**
Self-Care problem		7 (5%)	35 (21.74%)	48 (24.49%)		**<0.00001**
Sleep problem		41 (29.28%)	67 (41.61%)	76 (38.77%)		0.13841
Disorientation		33 (23.57%)	82 (50.93%)	81 (41.33%)		<**0.00001**
Incontinence		28 (20%)	44 (27.33%)	48 (24.49%)		**0.05862**
Loss of appetite		16 (11.43%)	40 (24.84%)	35 (17.86%)		**0.00919**
**Comorbid medical problems *n* (%)**						
Hypertension	19 (38%)	60 (42.86%)	79 (49.07%)	89 (45.41%)	247 (45.15%)	0.50628
Thyroid dys.	7 (14%)	22 (15.71%)	14 (8.69%)	23 (11.73%)	66 (12.06%)	0.29858
Diabetes Mellitus	9 (18%)	30 (21.43%)	36 (22.36%)	38 (19.39%)	113 (24.04%)	0.86285
CAD	12 (24%)	38 (27.14%)	35 (21.74%)	56 (28.57%)	141 (20.66%)	0.49531
Hyperlipidemia	9 (18%)	34 (24.28%)	35 (21.74%)	39 (19.90%)	117 (21.39%)	0.72651
Stroke	1 (2%)	6 (4.28%)	6 (3.73%)	12 (6.12%)	25 (4.57%)	0.54613
Current Smoker	7 (14%)	18 (12.86%)	8 (4.97%)	15 (7.65%)	48 (8.77%)	0.16899
No smoker	32 (64%)	88 (62.86%)	119 (73.91%)	138 (70.41%)	377 (68.93%)	-
Quit smoker	11 (22%)	34 (24.28%)	34 (21.12%)	43 (21.94%)	122 (22.30%)	-
Regular alcoholic	-	15 (10.72%)	13 (8.08%)	14 (7.14%)	42 (7.68%)	0.24199
Non-alcoholic	47 (94%)	122 (87.14%)	144 (89.44%)	172 (87.75%)	485 (88.66%)	-
Quit alcoholic	3 (6%)	3 (2.14%)	4 (2.48%)	10 (5.10%)	20 (3.66%)	-
Epilepsy	-	18 (12.86%)	22 (13.66%)	49 (25.0%)	89 (16.27%)	**0.00005**
Extrapyramidal symptoms	-	19 (13.57%)	18 (11.18%)	64 (32.65%)	102 (18.65%)	**<0.00001**
Family history of dementia *n* (%)	18 (36%)	61 (43.57%)	59 (36.64%)	82 (41.84%)	220 (40.22%)	0.55337
Family history of vascular disease	23 (46%)	73 (52.14%)	56 (34.78%)	76 (38.77%)	228 (41.68%)	**0.01463**

*p* values are significant.

Neuropsychiatric evaluation of study sample (Table 2) revealed that the scores for certain cognitive assessments varied across time. For instance, in the BDLAS test increased step by step in all groups suggesting functional deterioration in the process. Accordingly, EDLAS scores decreased step by step in all groups. As a screener test MMSE scores significantly decreased in all groups, suggesting a negative trend in cognitive performance over time. In contrast, the Digit Backward test scores remained relatively stable in non-Ad and MCI groups but decreased in AD group. However, digit backward test decreased in AD and non-AD sufferers but stable in MCI cases. While the WMT-recall scores remained relatively stable in non-AD cases (*p* > 0.05) but decreased in MCI cases (*p* = 0.0053), the scores for WMT-1 and WMT-2 increased from visit 1 to visit 2, suggesting a potential improvement in memory retention. However, a decline in performance was observed for WMT-3, indicating difficulty in recalling specific words. In terms of visual memory, the Visual Memory Score showed a decrease from 11 (0–11) (*n* = 48) during visit 1 to 8 (0–11) (*n* = 70) during visit 3. Similarly, the Visual Memory Recall score decreased from 8 (0–11) (*n* = 47) during visit 1 to 3 (0–11) (*n* = 67) during visit 3 supporting a decline in visual memory performance over time.

**Table 2:** Neuropsychiatric evaluation of the cases with AD.

	Prodromal dementia 140 (28.17%)	Alzheimer dementia 161 (32.39%)	Non-AD dementias 196 (39.44%)	Total 497 (100%)	*p*-value_B_
**BDLAS**					
Visit 1	1.20 ± 0.99	2.76 ± 1.97	2.15 ± 1.48	2.09 ± 1.67	<0.0001
	*n* = 125 (0–4.5)	*n* = 149 (0–8)	*n* = 172 (0–7.5)	*n* = 446 (0–8)	
Visit 2	1.63 ± 1.10	2.89 ± 2.23	2.30 ± 1.72	2.32 ± 1.85	0.00089
	*n* = 88 (0–6)	*n* = 114 (0–8)	*n* = 130 (0–8)	*n* = 332 (0–8)	
Visit 3	2.33 ± 1.64	4.12 ± 2.22	3.46 ± 2.14	3.43 ± 2.17	0.00026
	*n* = 64 (0–7.5)	*n* = 97 (0–8)	*n* = 95 (0–8)	*n* = 256 (0–8)	
*p*-value _W_	<0.0001	<0.0001	<0.0001		
**EDLAS**					
Visit 1	21.28 ± 3.07	15.19 ± 6.85	17.38 ± 6.17	17.75 ± 6.21	<0.0001
	*n* = 122 (8–23)	*n* = 144 (0–23)	*n* = 170(1–23)	*n* = 436 (0–23)	
Visit 2	19.78 ± 4.63	14.48 ± 7.98	16.18 ± 6.91	16.53 ± 7.09	0.00014
	*n* = 85 (3–23)	*n* = 113 (0–23)	*n* = 128 (0–23)	*n* = 326 (0–23)	
Visit 3	16.70 ± 6.86	9.69 ± 7.74	11.88 ± 7.80	12.25 ± 8.01	0.00083
	*n* = 63 (0–23)	*n* = 96 (0–23)	*n* = 93 (0–23)	*n* = 252 (0–23)	
*p*-value_W_	<0.0001	<0.0001	<0.0001		
**MMSE**					
Visit 1	27.28 ± 2.66	21.30 ± 6.43	22.59 ± 6.11	23.49 ± 5.99	<0.0001
	*n* = 140 (15–30)	*n* = 161 (0–30)	*n* = 196 (4–30)	*n* = 497 (0–30)	
Visit 2	26.74 ± 3.42	20.63 ± 7.74	21.59 ± 6.98	22.73 ± 6.94	<0.0001
	*n* = 140 (13–30)	*n* = 161 (0–30)	*n* = 196 (0–30)	*n* = 497 (0–30)	
Visit 3	23.06 ± 6.34	15.99 ± 8.17	17.82 ± 8.50	18.70 ± 8.32	0.00027
	*n* = 140 (3–30)	*n* = 161(0–30)	*n* = 196 (0–30)	*n* = 497 (0–30)	
*p*-value_W_	<0.0001	<0.0001	<0.0001		
**Digit forward**					
Visit 1	4 (2–7)	4 (0–6)	4 (2–7)	4 (0–7)	<0.0001
	*n* = 131	*n* = 136	*n* = 164	*n* = 431	
Visit 2	4 (3–7)	4 (0–10)	4 (0–7)	4 (0–10)	<0.0001
	*n* = 129	*n* = 126	*n* = 148	*n* = 403	
Visit 3	4 (0–7)	4 (0–7)	4 (0–7)	4 (0–7)	<0.0001
	*n* = 132	*n* = 121	*n* = 156	*n* = 409	
*p*-value_W_	0.8265	0.0155	0.2754		
**Digit backward**					
Visit 1	3 (0–5)	2 (0–5)	2 (0–6)	2 (0–6)	<0.0001
	*n* = 131	*n* = 132	*n* = 158	*n* = 421	
Visit 2	3 (0–6)	2 (0–5)	2 (0–6)	3 (0–6)	<0.0001
	*n* = 129	*n* = 121	*n* = 145	*n* = 395	
Visit 3	3 (0–7)	2 (0–5)	2 (0–5)	2 (0–7)	<0.0001
	*n* = 129	*n* = 116	*n* = 146	*n* = 391	
*p*-value_W_	0.1835	0.0089	0.0039		
**Calculation**					
Visit 1	5 (0–5)	5 (0–5)	5 (0–5)	5 (0–5)	<0.0001
	*n* = 128	*n* = 128	*n* = 159	*n* = 415	
Visit 2	5 (0–5)	5 (0–5)	5 (0–5)	5 (0–5)	0.0001
	*n* = 127	*n* = 113	*n* = 139	*n* = 397	
Visit 3	5 (0–5)	2 (0–5)	3 (0–5)	3 (0–5)	<0.0001
	*n* = 127	*n* = 111	*n* = 140	*n* = 371	
*p*-value_W_	<0.0001	<0.0001	<0.0001		
**Abstraction**					
Visit 1	3 (0–3)	3 (0–3)	3 (0–3)	3 (0–3)	<0.0001
	*n* = 131	*n* = 134	*n* = 162	*n* = 427	
Visit 2	3 (0–3)	3 (0–3)	3 (0–4)	3 (0–4)	<0.0001
	*n* = 130	*n* = 119	*n* = 146	*n* = 395	
Visit 3	3 (0–3)	3 (0–3)	3 (0–3)	3 (0–3)	<0.0001
	*n* = 130	*n* = 121	*n* = 146	*n* = 397	
*p*-value_W_	<0.0001	0.0004	<0.0001		
**WMT-1 (max:10)**					
Visit 1	3 (0–7)	2 (0–6)	2 (0–6)	2 (0–7)	<0.0001
	*n* = 124	*n* = 124	*n* = 154	*n* = 402	
Visit 2	3 (0–10)	2 (0–7)	2 (0–7)	3 (0–10)	<0.0001
	*n* = 127	*n* = 116	*n* = 132	*n* = 375	
Visit 3	3 (0–7)	1 (0–6)	2 (0–6)	2 (0–7)	<0.0001
	*n* = 127	*n* = 114	*n* = 146	*n* = 387	
*p*-value_W_	0.0391	0.0585	0.3806		
**WMT-2 (max:10)**					
Visit 1	5 (0–8)	3 (0–8)	3 (0–9)	3 (0–9)	<0.0001
	*n* = 125	*n* = 128	*n* = 156	*n* = 409	
Visit 2	4 (0–10)	3 (0–7)	3 (0–10)	4 (0–10)	<0.0001
	*n* = 127	*n* = 115	*n* = 131	*n* = 373	
Visit 3	4 (0–8)	2 (0–9)	3 (0–8)	3 (0–9)	<0.0001
	*n* = 128	*n* = 113	*n* = 150	*n* = 391	
*p*-value_W_	0.0015	0.0381	0.1562		
**WMT-3 (max:10)**					
Visit 1	5 (0–10)	3 (0–8)	4 (0–9)	4 (0–10)	<0.0001
	*n* = 126	*n* = 127	*n* = 155	*n* = 408	
Visit 2	5 (0–9)	3 (0–9)	4 (9–10)	4 (0–10)	<0.0001
	*n* = 126	*n* = 116	*n* = 130	*n* = 372	
Visit 3	5 (0–9)	2 (0–10)	3 (0–10)	3 (0–11)	<0.0001
	*n* = 128	*n* = 112	*n* = 151	*n* = 391	
*p*-value_W_	0.0211	0.0123	0.1222		
**WMT-recall (max:10)**					
Visit 1	3 (0–10)	0 (0–7)	0 (0–9)	1 (0–10)	<0.0001
	*n* = 122	*n* = 121	*n* = 153	*n* = 396	
Visit 2	3 (0–8)	0 (0–8)	0 (0–8)	1 (0–8)	<0.0001
	*n* = 123	*n* = 111	*n* = 127	*n* = 361	
Visit 3	2 (0–9)	0 (0–10)	0 (0–7)	0(0–9)	<0.0001
	*n* = 124	*n* = 50	*n* = 140	*n* = 371	
*p*-value_W_	0.0053	0.3479	0.0949		
**WMT-recog. (max:20)**					
Visit 1	17 (0–20)	14 (0–20)	15 (0–20)	16 (0–20)	<0.0001
	*n* = 122	*n* = 121	*n* = 154	*n* = 397	
Visit 2	18 (0–20)	13 (0–20)	14 (0–20)	15 (0–20)	<0.0001
	*n* = 126	*n* = 115	*n* = 132	*n* = 373	
Visit 3	16 (0–20)	10 (0–20)	12 (0–20)	14 (0–20)	<0.0001
	*n* = 127	*n* = 112	*n* = 148	*n* = 387	
*p*-value_W_	0.0036	0.0057	0.0064		
**BNT**					
Visit 1	14 (3–15)	11 (0–15)	13 (0–15)	13 (0–15)	<0.0001
	*n* = 128	*n* = 130	*n* = 163	*n* = 421	
Visit 2	14 (2–15)	12 (0–15)	13 (0–15)	13 (0–15)	<0.0001
	*n* = 124	*n* = 117	*n* = 143	*n* = 384	
Visit 3	13 (0–15)	10 (0–15)	11 (0–15)	12(0–15)	<0.0001
	*n* = 130	*n* = 115	*n* = 153	*n* = 398	
*p*-value_W_	0.0494	<0.0001	<0.0001		
**Comprehension**					
Visit 1	6 (1–6)	6 (0–6)	6 (0–6)	6 (0–6)	0.0168
	*n* = 77	*n* = 95	*n* = 86	*n* = 258	
Visit 2	6 (1–6)	6 (0–6)	6 (0–6)	6 (0–6)	<0.0001
	*n* = 87	*n* = 73	*n* = 69	*n* = 229	
Visit 3	6 (0–6)	3 (0–6)	3 (0–6)	4 (0–6)	<0.0001
	*n* = 90	*n* = 83	*n* = 101	*n* = 274	
*p*-value_W_	0.0463	<0.0001	0.0122		
**CDT**					
Visit 1	9 (0–10)	4 (0–10)	5 (0–10)	6 (0–10)	<0.0001
	*n* = 121	*n* = 126	*n* = 149	*n* = 396	
Visit 2	10 (0–10)	5 (0–10)	5 (0–10)	6 (0–10)	<0.0001
	*n* = 123	*n* = 110	*n* = 130	*n* = 363	
Visit 3	8 (0–10)	3 (0–10)	4 (0–10)	5 (0–10)	<0.0001
	*n* = 123	*n* = 113	*n* = 146	*n* = 382	
*p*-value_W_	0.1259	0.0169	0.1824		
**Visual memory score**					
Visit 1	11 (0–11)	5 (0–11)	8 (0–11)	8 (0–11)	<0.0001
	*n* = 48	*n* = 50	*n* = 36	*n* = 134	
Visit 2	10 (0–11)	4 (0–11)	7 (0–11)	7 (0–11)	<0.0001
	*n* = 49	*n* = 47	*n* = 43	*n* = 139	
Visit 3	8 (0–11)	5 (0–11)	7 (0–11)	7 (0–11)	<0.0001
	*n* = 70	*n* = 55	*n* = 76	*n* = 201	
*p*-value_W_	0.0797	0.0424	0.8920		
**Visual memory recall**					
Visit 1	8 (0–11)	1 (0–11)	3 (0–11)	4 (0–11)	<0.0001
	*n* = 47	*n* = 48	*n* = 35	*n* = 130	
Visit 2	7 (0–11)	0 (0–11)	2 (0–11)	2 (0–11)	<0.0001
	*n* = 47	*n* = 45	*n* = 40	*n* = 132	
Visit 3	3 (0–11)	0 (0–11)	0 (0–11)	0 (0–11)	<0.0001
	*n* = 67	*n* = 50	*n* = 71	*n* = 188	
*p*-value_W_	0.5284	0.0135	0.1225		
**GDS**					
Visit 1	2 (1–3)	3 (2–6)	3 (2–5)	3 (1–6)	<0.0001
	*n* = 140	*n* = 161	*n* = 195	*n* = 495	
Visit 2	3 (2–5)	4 (2–7)	4 (2–6)	4 (2–7)	<0.0001
	*n* = 140	*n* = 161	*n* = 195	*n* = 495	
Visit 3	3 (2–7)	5 (3–7)	5 (2–7)	4 (2–7)	<0.0001
	*n* = 140	*n* = 161	*n* = 195	*n* = 495	
*p*-value_W_	<0.0001	<0.0001	<0.0001		

BDLAS, Barthel Daily Living Activities Scale; EDLAS, Elderly Daily Living Activity Scale; MMSE, Minimental State Examination; WMT, Word memory test; BNT, Boston naming test; CDT, Clock Driving Test; GDS, Global Deterioration Scale. *p*-value_W_, *p*-value for within-group change; *p*-value_B_, *p*-value for differences between groups.

Laboratory evaluation of study sample showed that the prevalence of hippocampal atrophy across different grades. Grade 1 hippocampal atrophy was observed in 58.27% (*n* = 81) of the participants, followed by 35.26% (*n* = 49) with Grade 2 and 6.47% (*n* = 9) with Grade 3. Our data indicates that hippocampal atrophy was more prevalent in participants with prodromal dementia compared to those with Alzheimer’s dementia and non-Alzheimer’s dementias. As a representer of white matter hyperintensities The Fazekas grades reveals the distribution of participants across different grades. Grade 0 was present in 20.90% (*n* = 28) of the participants, followed by Grade 1 in 38.81% (*n* = 52), Grade 2 in 29.10% (*n* = 39), and Grade 3 in 11.19% (*n* = 15). These findings indicate a varied degree of white matter hyperintensities among the study participants, with Grade 2 being the most prevalent (refer to Table 3).

**Table 3:** Laboratory evaluation of the cases with AD.

	Prodromal dementia	Alzheimer dementia	Non-AD dementias	*P*
	140 (28.17%)	161 (32.39%)	196 (39.44%)	
Hippocampal atrophy *n* (%)				<0.00001
Grade 1	81 (58.27%)	28 (17.39%)	90 (45.92%)	
Grade 2	49 (35.26%)	81 (50.31%)	76 (38.77%)	
Grade 3	9 (6.47%)	52 (32.30%)	30 (15.31%)	
Fazekas *n* (%)				<0.00001
Grade 0	28 (20.90%)	-	35 (17.95%)	
Grade 1	52 (38.81%)	46 (28.57%)	62 (31.79%)	
Grade 2	39 (29.10%)	88 (54.66%)	60 (30.77%)	
Grade 3	15 (11.19%)	27 (16.77%)	38 (19.49%)	

In the study, various treatment approaches were utilized for different types of dementia (refer to Table 4). AChEIs treatment was administered to a significant proportion of patients across all dementia types. The percentage of patients receiving AChEIs was 85% for prodromal dementia, 94.41% for Alzheimer’s dementia, and 92.35% for non-AD dementias. The percentage of patients receiving memantine was 52.86% for prodromal dementia, 74.53% for Alzheimer’s dementia, and 68.37% for non-AD dementias. A significant percentage of patients received combination therapy, 50.71% for prodromal dementia, 73.29% for Alzheimer’s dementia, and 67.86% for non-AD dementias. The mean starting time for AChEIs treatment ranged from 37.55 ± 29.54 months for prodromal dementia to 32.53 ± 27.03 months for non-AD dementias. Similarly, the mean starting time for memantine treatment ranged from 52.63 ± 33.86 months for prodromal dementia to 40.80 ± 29.78 months for non-AD dementias. The percentages of patients receiving this combination therapy were 32.86% for prodromal dementia, 36.02% for Alzheimer’s dementia, and 63% for non-AD dementias in our dataset. In terms of the effects of therapies on the prognosis of dementia, our data revealed some interesting findings. Firstly, there was no significant difference in the starting time of Acetylcholinesterase inhibitors (AChEIs) between the groups (*p* = 0.1617). However, we observed that Memantine and combined therapy were initiated earlier in non-AD dementia cases compared to AD dementia cases and prodromal dementia (*p* = 0.0261 and *p* = 0.027089, respectively).

**Table 4:** Medical therapy profile of the cases with dementia.

	Prodromal dementia^*^	Alzheimer dementia	Non-AD dementias	Total	*p*
	140 (%)	161 (%)	196 (39.44%)	497 (100%)	
AChEIs, *n* (%)	119 (85.0%)	152 (94.41%)	181 (92.35%)	452 (90.94%)	0.1213
Mean starting time after the first symptom, mean ± SD	37.55 ± 29.54 119 (3–152)	36.87 ± 28.98 152 (3–129)	32.53 ± 27.03 181 (3–169)	35.31 ± 28.39 452 (3–169)	0.1617
Memantine, *n* (%)	74 (52.86%)	120 (74.53%)	134 (68.37%)	328 (65.99%)	0.00026
Mean starting time after the first symptom, mean ± SD	52.63 ± 33.86 74 (12–163)	46.16 ± 31.37 120 (8–144)	40.80 ± 29.78 134 (9–129)	45.43 ± 31.55 328 (8–163)	0.0261
Combination of AChEIs + Memantine, *n* (%)	71 (50.71%)	118 (73.29%)	133 (67.86%)	322 (64.79%)	0.00012
Mean starting time after the first symptom, mean ± SD	52.32 ± 33.41 71 (12–163)	46.13 ± 31.15 118 (8–144)	40.26 ± 29.08 133 (9–129)	45.07 ± 31.09 322 (8–163)	0.02708
EGb761 like Gingko extract, *n* (%)	88 (54.66%)	107 (54.59%)	110(78.57%)	305	0.00001
Combination of AChEIs + Memantine + EGb761, *n* (%)	46 (32.86%)	58 (36.02%)	62 (63%)	166 (33.40%)	0.67286

AChEIs, Cholinesterase inhibitors; ^*^Prodromal dementia cases stated the same name even after they turned to clinical stages of dementia.

**Figure 2:**
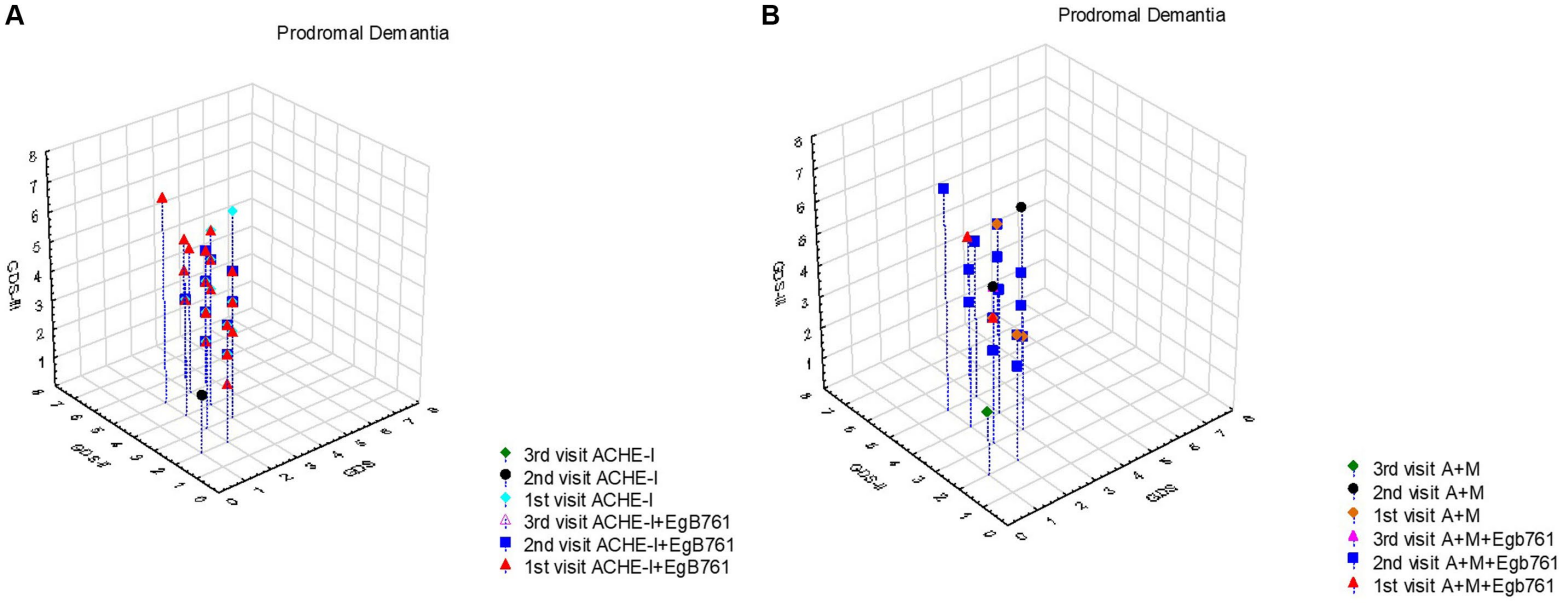
The effect of mentioning drugs on the progression prodromal dementia (MCI). **(A)** It appears that prodromal dementia patients had GDS scores of 3 and below at first admission, and most of them were given additional EGb761 like *Gingko* extract with ACHE at first admission. Following visits these cases commonly progressed to clinical dementia and GDS scores were maximal 5 at the second visit and 6 at third visit. When evaluated together with Table 5, it is seen that the prognosis of those who were given only ACHE and those who were added EGb761 like *Gingko* extract were similar. **(B)** It is seen that the combined treatment rate is extremely low in Prodromal dementia patients at the first admission and EGb761 like *Gingko* extract is usually given together with the combined treatment at the second visit because of they were progressed to clinical stage of dementia. In this group, it is seen that the first admission GDS scores of these patients were 3 and below, and the maximum value of the GDS scores, which was 5 in the second visit, was 7 in the third visit. When evaluated together with Table 5, it is seen that the prognosis of those who only received combined treatment and those who added EGb761 like *Gingko* extract was similar.

Our study provides valuable insights into the treatment patterns and progression of prodromal dementia patients, shedding light on the impact of additional EGb761 like *Gingko* extracts with ACHE and the utilization of combined treatment as shown in Figure 2. Figure 2A reveals that prodromal dementia patients, initially presenting with GDS scores of 3 or below, commonly received additional EGb761 like *Gingko* extract with ACHE at first admission. Subsequent visits showed a progression to clinical dementia, with GDS scores peaking at 5 during the second visit and reaching 6 during the third visit. Notably, when considering Table 5, the prognosis of patients receiving only ACHE and those with added EGb761 like *Gingko* extract appeared similar. Figure 2B highlights the low utilization of combined treatment among prodromal dementia patients at first admission. However, at the second visit, EGb761 like *Gingko* extract was frequently administered alongside the combined treatment due to the patients’ transition to the clinical dementia stage. Interestingly, patients’ initial GDS scores were 3 or below, with maximum scores of 5 in the second visit and 7 in the third visit. Analysis with Table 5 indicates comparable prognoses between patients receiving only combined treatment and those with added EGb761 like *Gingko* extract.

**Table 5:** The effects of therapies on the prognosis of dementia subtypes.

	Prodromal dementia		*p*-value	Alzheimer dementia		*p*-value	Non-AD dementias		*p*-value
	*n* = 140			*n* = 161			*n* = 196		
	Slow	Fast		Slow	Fast		Slow	Fast	
AChEIs	β = 1.72586		0.0608	β = 0.86467		0.2917	β = 1.56844		0.0238
	59 (77.63)	60 (93.75)		78 (92.86)	74 (96.10)		81 (87.10)	100 (97.09)	
AChEIs starting time	39.44 ± 31.59	35.68 ± 27.51	0.4902	39.52 ± 29.35	34.07 ±28.51	0.2471	28.23± 21.35	36.01 ± 30.54	0.0541
Memantine	β = 2.43514		<0.0001	β = 1.64355		0.0004	β = 1.46772		0.0003
	23 (30.26)	51 (79.69)		53 (63.10)	67 (87.01)		52 (55.91)	82 (79.61)	
Memantine starting	56.17 ± 34.78	51.04 ± 33.66	0.5497	51.04 ± 32.36	42.29 ± 30.25	0.1303	38.65 ± 28.31	42.15 ± 30.77	0.5088
Combined	β = 2.27617		<0.0001	β = 1.38712		0.0012	β = 1.40195		0.0005
	22 (28.95)	49 (76.56)		53 (63.10)	65 (84.42)		52 (55.91)	81 (78.64)	
Combine starting	53.27 ± 32.63	51.89 ± 34.07	0.8740	51.04 ± 32.36	42.12 ± 29.78	0.1226	38.65 ± 28.31	41.29 ± 29.69	0.6110
AChEIs + EGb761 like Gingko extract	β = 0.30426		0.2930	β = −0.62153		0.0389	β = 0.01913		0.9385
	48 (63.16)	41 (64.06)		51 (60.71)	28 (36.36)		47 (50.54)	46 (44.66)	
Combined + EGb761 like Gingko extract	β = 1.10699		<0.0001	β = 0.29216		0.1922	β = 0.43531		0.0308
	14 (18.42)	32 (50.00)		34 (40.48)	24 (31.17)		29 (31.18)	33 (32.04)	

**Figure 3:**
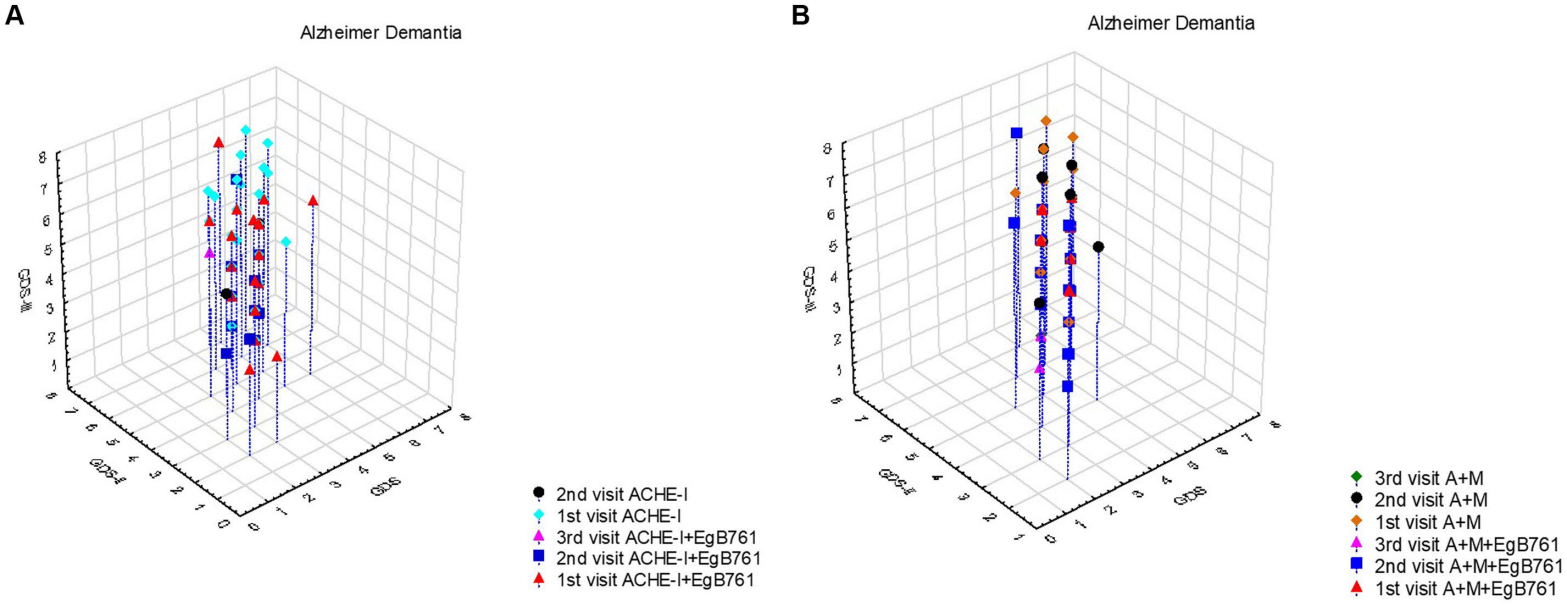
The effect of mentioning drugs on the progression of Alzheimer Dementia. **(A)** It was observed that the prognosis was slow in Alzheimer’s dementia patients who were started on the drug at the first application or at the second visit and given additional EGb761, and these patients had a GDS score of 5 and below at the first admission. It is seen that starting drugs and EGb761 at an earlier stage also slows down the progression. It is seen that the prognosis in patients who are started on the drug but not given EGb761 at the first admission is faster than the patients who are started on the drug and EGb761 at the first application. The results in Table 5 also support this. Adding EGb761, such as *Gingko* extract, to AChEIs decreased the progression of AD dementia (β = −0.705, *p* = 0.014). **(B)** It was observed that the number of patients receiving EGb761 in addition to the combined treatment was relatively less and did not significantly affect the prognosis in Alzheimer’s patients. The maximum GDS score was 6 at the first visit, and 7 at the second and third visit. This figure was also prominently revealed in cases with AChEIs, including combined therapy (β = −0.607, *p* = 0.028).

Our results revealed that highlight the importance of early drug initiation and the potential benefit of adding EGb761 like *Gingko* extract, but also suggest that the number of patients receiving this additional treatment is relatively low and does not significantly affect the prognosis in Alzheimer’s patients as shown in Figure 3. Figure 3A provides valuable insights into the prognosis of Alzheimer’s dementia patients based on the timing of drug initiation and the addition of EGb761 like *Gingko* extract. Patients who started the drug at the first application or at the second visit and received additional EGb761 exhibited a slower progression of the disease. These patients had GDS scores of 5 and below at the first admission, indicating a less severe condition. Furthermore, initiating the drug and adding EGb761 like *Gingko* extract at an earlier stage showed a further slowdown in disease progression. Notably, patients who started the drug but did not receive EGb761 like *Gingko* extract at the first admission had a faster prognosis compared to those who received both treatments initially. These findings are supported by the results presented in Table 5, indicating that adding EGb761 like *Gingko* extract to AChEIs decreased the progression of Alzheimer’s dementia. In Figure 3B, we observed that the number of patients receiving EGb761 like *Gingko* extract in addition to the combined treatment was relatively low. However, this additional treatment did not significantly impact the prognosis of Alzheimer’s patients. The maximum GDS score reached 6 during the first visit and increased to 7 during the second and third visits. Importantly, this pattern was predominantly observed in cases involving AChEIs, including combined therapy. The coefficient of −0.607 suggests a potential association between the use of AChEIs and the disease progression, although the value of p was not statistically significant.

**Figure 4:**
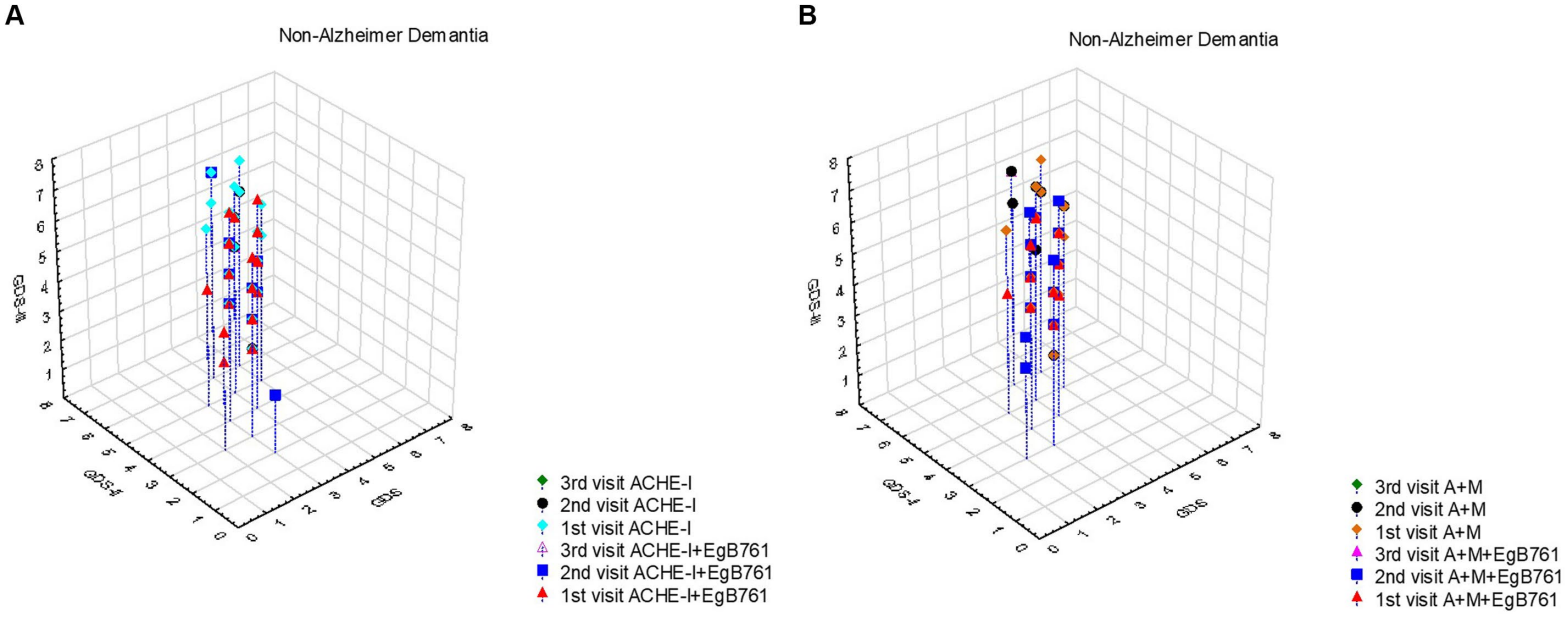
The effect of mentioning drugs on the progression Non-Alzheimer Dementia. **(A)** In patients with a slow non-Alzheimer dementia prognosis, the rate of those who started EGb761 like Gingko extract with the drug at the first visit is higher than those with a fast prognosis. In addition, it is seen that the prognosis is faster in those who only start ACHE at the first visit. The maximum GDS score was 5 at the first visit, 6 at the second visit, and 7 at the third visit. **(B)** Progression monitored by GDS was slower in patients with non-Alzheimer dementia who switched to combination therapy early and in whom EGb761-like Gingko extracts were used in combination.

In the aspect of non-Alzheimer dementia, we showed that a higher proportion of patients who started EGb761 like *Gingko* extract alongside the drug at the first visit exhibited a slower prognosis compared to those with a fast prognosis (Figure 4). Additionally, it is observed that patients who solely initiated AChEIs at the first visit had a faster prognosis. The maximum GDS score recorded was 5 during the first visit, 6 during the second visit, and 7 during the third visit.

Analyzing the results of trajectory analysis (refer to Table 5), we found no significant effect of AChEIs on the prognosis of AD (*p* = 0.2917). However, cases that received AChEIs in combination with EGb761 like *Gingko* extract showed slower progression compared to those using AChEIs alone (β = −0.62153, *p* = 0.0389). It is noteworthy that 60.71% of cases with slow progression reported regular use of EGb761 like *Gingko* extract. Additionally, our study demonstrated a favorable effect of Memantine on disease progression (*p* = 0.0004), with a significant proportion (87%) of patients showing effective dose usage and experiencing fast progression. Similarly, 84.42% of our sample had combined therapy, further supporting its positive impact on disease progression. However, the addition of EGb761 like Gingko extract to combined therapy did not show any significant effect on disease progression (*p* = 0.1922). No significant difference was observed in the starting time of medication use among the groups.

On the other hand, the use of AChEIs had a significant effect on the progression of non-AD dementia (*p* = 0.0238), with almost 97.09% of cases in the fast prognostic group reporting regular use of AChEIs. Furthermore, the addition of memantine to the regimen had a significant effect on prognosis (*p* = 0.0003), with 79.61% of the fast prognostic group reporting regular use of memantine. In the non-AD group, combined therapy showed a significant effect on prognosis (*p* = 0.0005), with 78.64% of non-AD dementia cases with a fast prognosis receiving combined therapy. Although non-AD dementia cases did not show an effect of adding EGb761 like *Gingko* extract to AChEIs, there was a significant effect when added to combined therapy (*p* = 0.0308). Overall, our results indicated that adding EGb761 like *Gingko* extract to the regimen decreased the progression rate of non-AD dementia cases (β = 0.43531 vs. β = 1.40195).

Regarding Mild Cognitive Impairment (MCI), our data demonstrated no significant effect of AChEIs on the progression to clinical dementia in general (β = 1.72586, *p* = 0.0608). Moreover, adding EGb761 like *Gingko* extract to AChEIs showed a proportional decrease in progression, although not statistically significant (β = 0.30426, *p* = 0.2930). Interestingly, we found that 63.16% of MCI cases with slow progression were managed with EGb761 like *Gingko* extract. For cases that started as MCI but progressed to clinical dementia, receiving memantine in the early or moderate stages of the disease had a significant favorable effect on prognosis (*p* < 0.0001). Specifically, 79.69% of memantine-started MCI cases and 76.56% of cases receiving combined therapy had a fast prognosis, demonstrating statistical significance.

## 4 Discussion

In this study, we aimed to investigate the impact of pharmacological interventions on the progression of dementia, with a specific focus on acetylcholinesterase inhibitors (AChEIs), memantine, and EGb761 like *Gingko* extract. These drugs have shown efficacy in improving cognitive and overall function in individuals with different levels of AD and non-AD dementias, some of them are affective in MCI with limited references. Our analysis included a total of 547 participants from our 211 months follow-up duration dataset, consisting of healthy controls, prodromal dementia (MCI), Alzheimer’s disease, and non-Alzheimer dementias. In terms of treatment approaches, a significant proportion of patients across all dementia types received acetylcholinesterase inhibitors (AChEIs), memantine, or combination therapy. The starting time for these treatments varied, with memantine and combination therapy initiated earlier in non-AD dementia cases compared to AD dementia cases and progressed from the prodromal dementia. Furthermore, our trajectory analysis indicated that the combination of AChEIs with EGb761 like *Gingko* extract showed a slower progression compared to AChEIs alone in prodromal dementia and AD cases. Overall, our findings suggest that pharmacological interventions, particularly the use of AChEIs and memantine, can have an impact on cognitive function and overall function in individuals with dementia. The combination of AChEIs with EGb761 may have additional benefits in slowing disease progression in AD cases.

Consensus diagnostic criteria for MCI are available, making early recognition and accurate classification of MCI subtypes possible. MCI can be identified in a primary care setting. Further corroboration and differential diagnosis should be done at specialist level. There are biological and epidemiological risk factors of disease progression from MCI to dementia have been identified, including apolipoprotein E4 (APOE-e4), depression, loneliness, hearing impairment, diabetes, hypertension, older age, female gender, and stronger cognitive impairment as reflected by lower MMSE score or higher AD assessment scale cognitive subscale (ADAS-cog) score (13). Our data suggested adding hippocampal atrophy and WMIs grading by Fazekas and importance of WMTs specifically.

Individuals with MCI have an increased risk of developing dementia (10%–15% per year), and early intervention may slow the progression of cognitive decline and delay the onset of dementia. Mixed pathologies are the rule in MCI, thus a multi-target treatment approach is a rational strategy. Promising evidence has been generated for multi-domain interventions. Limited evidence is available for different pharmacological classes that have been investigated in MCI clinical trials (e.g., acetylcholinesterase inhibitors). EGb 761®like *Gingko* extract improved symptoms in some clinical trials; it is the only pharmacological treatment recommended in existing guidelines for the symptomatic treatment of MCI (14, 15). There is currently no effective pharmacological intervention for MCI. Potential agents for MCI treatment include AChEIs, glutamate receptor modulators, antioxidants, anti-inflammatory drugs, nootropics, immunomodulators, mainly amyloid antibodies, secretase inhibitors and *Ginkgo biloba* (16). Main stakeholder in pharmacological approach is Cholinesterase inhibitors (AChEIs) which suggested that early intervention with AChEIss may also be beneficial for individuals with MCI supporting long term RCTs (17). Current symptomatic agents with a single mode of action may only have limited impact on mixed pathologies, whereas therapy with a multi-factorial mode of action (e.g., EGb 761VR) promises to be a more rational choice in this context. Personalized risk predictions in patients with MCI based on differential biomarker constellations may be possible in the future (18). The results of our study on the management of MCI indicate that the use of pharmacological interventions, specifically AChEIs (if progresses the clinical stage) memantine, and EGb761 like *Gingko* extract, can have an impact on the progression of MCI. These drugs have shown efficacy in enhancing cognitive function and overall function in individuals with MCI, potentially delaying or preventing the transition to dementia. Our findings reveal that a significant proportion of MCI patients received AChEIs treatment (85%) from the first diagnosis, memantine (52.86%), or combination therapy (50.71%) in the process. The starting time for AChEIs treatment ranged from 37.55 to 32.53 months, while the starting time for memantine treatment ranged from 52.63 to 40.80 months. Notably, memantine and combined therapy were initiated earlier in non-AD dementia cases compared to AD dementia cases and prodromal dementia. Regarding the effects of therapies on the prognosis of MCI, the use of AChEIs alone did not show a significant difference in the progression of AD. However, cases that received AChEIs in combination with EGb761 like *Gingko* extract demonstrated a slower progression compared to those using AChEIs alone. This suggests that the addition of EGb761 like *Gingko* extract to AChEIs may have a synergistic effect in managing MCI and potentially slowing down disease progression in a long term (maximum 211 months) comprehensive follow-up data.

Symptomatic management of Alzheimer’s disease typically involves a multidisciplinary approach that combines pharmacological and non-pharmacological interventions. Pharmacological treatments focus on targeting cognitive symptoms and may include acetylcholinesterase inhibitors (AChEIs) such as donepezil, rivastigmine, and galantamine. These medications work by increasing the availability of acetylcholine in the brain, which can help improve cognitive function, memory, and daily living activities to some extent. Studies have suggested that early intervention with AChEIs may be beneficial for individuals with AD. A meta-analysis of randomized controlled trials (RCTs) investigated the effects of early intervention with AChEIs on the progression of cognitive decline in patients with AD. The meta-analysis included 14 RCTs with a total of 7,232 participants, and found that treatment with AChEIs was associated with a significant reduction in the decline of cognitive function compared to placebo (19). Our long-term follow-up data revealed that AChEIs were commonly prescribed to a significant proportion of our patients with AD but the use of alone did not significantly impact the prognosis. However, the combination of AChEIs and EGb761 showed a potential benefit in slowing down disease progression compared to AChEIs alone in individuals with Alzheimer’s disease.

Another medication used in the management of moderate to severe Alzheimer’s disease is memantine, an NMDA receptor antagonist. Memantine helps regulate glutamate activity and may alleviate symptoms such as confusion, agitation, and aggression (20). Even that Cholinesterase inhibitors and memantine have been approved for management of AD, but there has been no consensus about the choice of various types and doses of drugs at different stages. A network meta-analysis of 41 randomized controlled trials showed that galantamine 24 mg daily (− 0.50, − 0.61 to −0.40), and donepezil 10 mg daily (− 0.40, − 0.51 to −0.29) were probably the most effective agents on cognition for mild to moderate AD, and memantine 20 mg combined with donepezil 10 mg (0.76, 0.39 to 1.11) was recommended for moderate to severe patients. Memantine showed the best profile of acceptability. Rivastigmine transdermal 15-cm2 patch was the best optional treatment both in function and global changes (21). Our moderate and severe AD patient’s follow-up data suggested that there was no significant effect on the progression of AD. However, combination therapy with AChEIs and EGb761 like *Gingko* extract has slower disease progression was observed compared to AChEIs alone. On the other hand, Memantine showed a favorable effect on disease progression, with a significant proportion of patients experiencing fast progression. Moreover, Combined therapy (AChEIs and Memantine) showed a positive impact on disease progression. But, addition of EGb761 like *Gingko* extract to combined therapy did not show a significant effect on disease progression. Regarding study design most of the early and early-moderate stages of our cases stated under the title of prodromal dementia and progressed there accordingly.

Aside from AD, few other dementia etiologies have approved pharmacologic treatments for cognitive symptoms, and no disease specific treatments exist for Lewy body disease or frontotemporal dementia. In addition to AD, rivastigmine has also received approval for Parkinson disease dementia. There are currently no FDA-approved drugs for MCI and studies of acetylcholinesterase inhibitors failed to show benefit in this population (22). Even that there are neither a consensus nor evidence based compatible data, mentioning drugs still are options for non-AD dementias (23). Our non-AD dementia cases in which are commonly VaD and LBD/PDD cases showed that regular use of AChEIs had a significant effect on slowing disease progression. Addition of Memantine to the regimen also had a significant effect on prognosis, with a significant proportion of patients in the fast prognostic group reporting regular use of memantine. In the aspect of combined therapy, our data showed a significant effect on prognosis in non-AD dementia cases. Addition of EGb761 to combined therapy also showed a significant effect on prognosis, decreasing the progression rate of non-AD dementia cases.

EGb761 is a standardized extract of *Ginkgo biloba*, which has been developed in the 1960’s in Germany by Dr. Wilmar Schwabe and then proposed for some mental illness including dementia. Previously traditional Chinese medicine used for seeds rather than leaves some medical issues. The effect of EGb761 like *Gingko* extract on different subtypes of dementia has been studied, including Alzheimer’s disease (AD), vascular dementia (VaD), and mixed dementia (MD). The effect of EGb761 on cognitive function in patients with AD has been studied in several randomized controlled trials (RCTs). A meta-analysis of 11 RCTs found that EGb761 was associated with a significant improvement in cognitive function, particularly in the domains of attention and memory, compared to placebo (24). Final reports also suggested the role on the decreasing progression from MCI to clinical stages of dementia (25). In this study, patients with started MCI, addition of EGb761 like *Gingko* extract to AChEIs showed a proportional decrease in progression, although not statistically significant. It is worth mentioning that a significant proportion of MCI cases with slow progression were managed with EGb761. EGb761In AD Dementia patients; addition of EGb761 like *Gingko* extract to combined therapy (AChEIs and Memantine) did not show a significant effect on disease progression. It is important to note that our study did not find a significant difference in the starting time of medication use among the groups. In the aspect of Non-AD Dementia, addition of EGb761 to AChEIs did not show a significant effect on disease progression. Addition of EGb761 like *Gingko* extract to combined therapy showed a significant effect on prognosis, decreasing the progression rate of non-AD dementia cases. Our findings indicate that the addition of EGb761 to the treatment regimen had varying effects depending on the type of dementia. In non-AD dementia, the addition of EGb761 like *Gingko* extract to combined therapy showed a significant positive impact on prognosis, whereas in AD dementia and MCI, its effects were not statistically significant. However, it is essential to consider these findings in the context of the specific study, and further research and examination of existing literature are needed to draw broader conclusions about the role of EGb761 like *Gingko* extract in dementia management. As summarized in Table 6 the findings from various studies on the efficacy and safety of EGb 761 like *Ginkgo biloba* extracts in patients with different types of dementia. The studies evaluated the impact of EGb761 like *Ginkgo biloba* extracts on cognitive function, neuropsychiatric symptoms, and disease progression. The results indicate that EGb761 like *Ginkgo biloba* extracts at different doses showed potential benefits in improving cognitive function and neuropsychiatric symptoms in patients with dementia. Additionally, the combination of EGb761 like *Ginkgo biloba* extracts with AChEIs demonstrated a slower disease progression in individuals with MCI and especially in early stages of AD, but not limited to AD. The studies also reported a favorable safety profile for EGb761 like *Ginkgo biloba* extracts. However, one study did not find significant efficacy in improving cognitive function in patients with mild to moderate dementia of the Alzheimer’s type. Further research is needed to confirm the efficacy and the optimum dose and combination timing in larger trials.

**Table 6:** Summary of some references mentioning the effect of EGb761 like *Gingko* extract and AChEIs, Memantine or combination of both drugs.

Author, Year	MCI	AD	Non-AD dementias	EGb761 like *Gingko* extracts	AChEIs	Memantine	Notes
	*n* (%)	*n* (%)	*n* (%)				
García-Alberca et al. (26)	133 (100)	-	-	54 (40.6)	31 (23.3)	-	Provides evidence for the benefits of combining EGb761with AChEIs in MCI patients
				48 (36.1)			
Howard et al. (27)	-	295 (100)	-	-	146 (49.4)	76 without AChEIs	Donepezil and memantine may provide cognitive and functional benefits in moderate to severe Alzheimer’s disease, the trial found no evidence that these drugs significantly delay nursing home placement.
						73 with AChEIs	
Herrschaft et al. (28)		402 (100)		164 patients with EGb 761,240 mg/day or placebo for 24 weeks	-	-	*Ginkgo biloba* extract EGb761 at a dose of 240 mg/day can improve neuropsychiatric symptoms and cognition in patients with dementia, with a favorable safety profile.
Ihl et al. (29)	-	410 (100)	-	202 patients with EGb761 240 mg/day and 202 patients with placebo for 24 weeks	-	-	*Ginkgo biloba* extract EGb761administered at a dosage of 240 mg has demonstrated the potential to enhance neuropsychiatric symptoms and cognitive function in individuals with dementia, exhibiting a positive safety profile
Kanowski et al. (30)	-	156 (100)	-	79 patients with EGb761 240 mg/day and 77 patients with placebo for 24 weeks	-	-	*Ginkgo biloba* extract EGb761at a dose of 240 mg daily can slow cognitive decline and functional deterioration in patients with mild to moderate dementia, with a favorable safety profile.
Le Bars et al. (31)		236 (76.4)	73 (23.6)	155 patients with EGb761240 mg/day and 154 patients with placebo for 26 weeks	-	-	*Ginkgo biloba* extract EGb761 at a dose of 240 mg daily can slow cognitive decline and functional deterioration in patients with dementia, with a favorable safety profile.
Napryeyenko et al. (32)	-	391 (100)		196 patients with EGb761 240 mg/day and 195 patients with placebo for 22 weeks	-	-	*Ginkgo biloba* extract EGb761 at a dose of 240 mg daily can improve neuropsychiatric symptoms and cognition in patients with dementia, with a favorable safety profile.
Schneider et al. (33)	-	513 (100)	-	170 patients with EGb761 240 mg/day and 169 patients with EGb761 120 mg/day and 174 patients with placebo for 26 weeks	-	-	*Ginkgo biloba* extract EGb761 at doses of 120 mg or 240 mg daily improved cognitive function in patients with mild to moderate dementia of the Alzheimer’s type
Our study	140 (28.2)	161 (32.4)	196 (39.4)	305 (61.36) patients with EGb761 160–240 mg/day and 166 (33.4) patients with combination of AChEIs/Mematine and EGb761 like *Gingko* extract 160–240 mg/day	452 (90.94)	322 (64.79) with AChEIs 6 (1.2) (without AChEIs)	Combination therapy of EGb761 like *Gingko* extract with AChEIs and/or Memantine demonstrated a slower progression compared to AChEIs and/or Memantine alone in individuals with prodromal dementia (MCI) and early stages of AD

### 4.1 Implications of the study are as follows

- Pharmacological interventions, including acetylcholinesterase inhibitors (AChEIs), memantine, and EGb761 like *Gingko* extract, have shown efficacy in improving cognitive function and overall function in individuals with MCI, AD, and non-Alzheimer dementias. This highlights the potential for these drugs to delay the progression of cognitive decline and prevent the transition to dementia.
- Combination therapy with AChEIs and EGb761 may have additional benefits in slowing disease progression in individuals with prodromal dementia and AD. This suggests that the addition of EGb761 to AChEIs may have a synergistic effect in managing MCI and AD.
- The use of AChEIs alone did not significantly impact the prognosis of AD. However, the combination of AChEIs and EGb761 like *Gingko* extract showed potential benefits in slowing down disease progression compared to AChEIs alone in individuals with AD.
- Memantine, an NMDA receptor antagonist, has a favorable effect on disease progression in moderate to severe AD. Combined therapy with AChEIs and memantine also showed a positive impact on disease progression.
- EGb761 like standardized extract of *Ginkgo biloba*, has been associated with cognitive improvement in patients with AD. It has the potential to enhance attention and memory functions.
- Regular use of AChEIs and memantine had significant effects on slowing disease progression in non-AD dementias, such as vascular dementia (VaD) and Lewy body dementia (LBD)/Parkinson’s disease dementia (PDD). Addition of EGb761 to the combined therapy further decreased the progression rate of non-AD dementia cases.
- The study highlights the need for personalized risk predictions based on differential biomarker constellations in patients with MCI. This approach could help tailor interventions and optimize outcomes.
- The study samples are comprehensive and do not have any bias in terms of patient or therapy selection, including the exporting of data and analysis. This aspect of the study has a favorable effect on the results.
- To the best of our knowledge, this is one of the most common follow-up duration (max 211 months) natural history studies.
- The findings contribute to the existing evidence on the potential benefits of pharmacological interventions for cognitive symptoms in dementia. However, further research, including long-term randomized controlled trials, is needed to establish the efficacy and optimal treatment approaches for different dementia subtypes.

### 4.2 Limitations

Our study has several limitations needs to be addressed:

- Sample Size: The study included a total of 547 participants out of 3,547 cases, which may be considered relatively small for drawing generalizable conclusions about the impact of pharmacological interventions on dementia progression. A larger sample size would have provided more statistical power and increased the reliability of the findings.
- Study Design: The study relied on a retrospective analysis of data collected over a 211-month follow-up period. Retrospective studies are prone to biases and limitations, including recall bias and incomplete or missing data. A prospective study with standardized protocols and assessments would have provided more robust evidence.
- Treatment Variability: The study mentioned that a significant proportion of patients received different pharmacological interventions, including AChEIs, memantine, and combination therapy. The specific dosages, duration of treatment, and adherence rates were not clearly described. The lack of uniformity in treatment regimens limits the ability to draw precise conclusions about the effectiveness of specific interventions.
- Lack of Randomization: The study did not mention randomization or allocation concealment methods, which are important for reducing selection bias and ensuring balanced treatment groups. The absence of randomization introduces the potential for confounding factors that may influence the observed outcomes.
- Generalizability: The study population consisted of participants from a single center or a specific geographic region, which may limit the generalizability of the findings to other populations or healthcare settings. Factors such as cultural differences, access to healthcare, and variations in treatment practices may affect the applicability of the results to broader populations.
- Lack of Control Group: Even that we had a healthy control group followed by mentioning duration and characteristics, the study did not include a control group, such as individuals with dementia who did not receive any pharmacological interventions. Without a control group, it is challenging to determine the true effects of the treatments being studied and assess whether the observed outcomes are solely attributable to the interventions or influenced by other factors.
- Potential Confounding Variables: The study mentioned several risk factors associated with disease progression, such as apolipoprotein E4 (APOE-e4), depression, loneliness, hearing impairment, diabetes, hypertension, older age, female gender, and cognitive impairment. However, the analysis did not account for these potential confounding variables, which could have influenced the results and introduced bias into the findings.
- Multiple Outcome Measures: The study mentioned improvements in cognitive and overall function as the primary outcomes of interest. However, it did not provide specific details about the assessment tools used or whether the observed improvements were clinically significant. The lack of standardized outcome measures makes it difficult to compare the findings with other studies and evaluate the true impact of the interventions.

## 5 Conclusion

In conclusion, this comprehensive analysis sheds light on the complex nature of dementia progression and the potential role of pharmacological interventions in managing the condition. Our study highlights the potential benefits of pharmacological interventions, specifically AChEIs, memantine, and EGb761 like *Gingko* extract, in the management of dementia. These drugs have shown efficacy in improving cognitive function and overall function in individuals with MCI, AD, and non-AD dementias. The combination of AChEIs and/or Memantine with EGb761 like *Gingko* extract may have additional benefits in slowing disease progression, particularly in AD cases. Personalized risk predictions and consideration of biomarker constellations can further enhance treatment decisions and prognosis assessment. However, further research is needed to validate and optimize the use of these drugs in dementia management.

## Data availability statement

The data analyzed in this study is subject to the following licenses/restrictions: only available to registered doctors. Requests to access these datasets should be directed to http://www.epikriz.com/index.aspx.

## Ethics statement

The studies involving humans were approved by Toros University Ethical Committee decision no: 2023/28, date: 10.03.2023. The studies were conducted in accordance with the local legislation and institutional requirements. The participants provided their written informed consent to participate in this study.

## Author contributions

AÖ: data collection, conception, design, and revision of the article. RG and NÖ: data collection. AÖ, RG, NÖ, and BT: analysis and drafting of the article. AÖ: revision of the article and approval of the final version. All authors contributed to the article and approved the submitted version.

## Funding

The authors declare that this study received funding from Abdi İbrahim Pharmaceuticals. The funder was not involved in the study design, collection, analysis, interpretation of data, the writing of this article, or the decision to submit it for publication.

## Footnotes

^1^ www.epikriz.com/dementiadataset
